# Preparation and Characterization of PLA-PEG-PLA/PEI/DNA Nanoparticles for Improvement of Transfection Efficiency and Controlled Release of DNA in Gene Delivery Systems

**Published:** 2019

**Authors:** Amin Amani, Toraj Kabiri, Samira Shafiee, Aliasghar Hamidi

**Affiliations:** a *Biotechnology Research Center, Department of Medicinal Chemistry, Faculty of Pharmacy, Tabriz University of Medical Sciences, Tabriz, Iran. *; b *Department of Agronomy and Plant Breeding, Faculty of Agriculture, University of Mohaghegh Ardabili, Ardabil, Iran.*; c *Department of Medicinal Chemistry, Faculty of Pharmacy, Tabriz University of Medical Sciences, Tabriz, Iran.*

**Keywords:** PLA-PEG-PLA, Polyethylenimine, Gene delivery, Cytotoxicity, DNA release

## Abstract

Tri-block poly (lactide) poly(ethylene glycol) poly(lactide) (PLA–PEG–PLA) copolymers are among the most attractive nano-carriers for gene delivery into mammalian cells, due to their biocompatibility and biodegradability properties. However, the low efficiency of the gene delivery by these copolymers is an obstacle to gene therapy. Here, we have investigated nanoparticles formulated using the polyethylenimine (PEI) associated with PLA-PEG-PLA copolymer for efficient DNA encapsulation and delivery. PLA-PEG-PLA/DNA and PLA-PEG-PLA/PEI/DNA nanoparticles with different concentrations of PEI were prepared by the double emulsion-solvent evaporation technique. PLA-PEG-PLA/PEI/DNA were characterized for particle size, zeta potential, morphology, biocompatibility, DNA protection, DNA release, and their ability for gene delivery into MCF-7 cells. We found that enhancing the mass ratio of PEI: (PLA-PEG-PLA) (w/w%) in the PLA-PEG-PLA/PEI/DNA nanoparticles results in an increase in particles size, zeta potential, encapsulation efficiency, and DNA release. The electrophoretic analysis confirmed that the PLA-PEG-PLA and PLA-PEG-PLA/PEI could protect DNA from ultrasound damage and nuclease degradation. MTT assay showed that the PLA-PEG-PLA/PEI/DNA had low cytotoxicity than PEI complexes. The potential of PLA-PEG-PLA/PEI/DNA nanoparticles with different concentrations of PEI as a non-viral gene delivery vector for transferring pEGFP-N1 to MCF-7 cells was examined by fluorescent microscopy and flow cytometry. The flow cytometry analysis revealed that by increasing the mass ratio of PEI: (PLA-PEG-PLA) (w/w%) in PLA-PEG-PLA/PEI/DNA nanoparticles, the efficiency of the gene delivery into MCF-7 cells was improved. The results also demonstrated that PLA-PEG-PLA/PEI/DNA nanoparticles in the serum medium improved the efficiency of gene delivery more than two-fold, compared to PEI/DNA complex.

## Introduction

Advances in nanotechnology and biotechnology have brought nano-carrier mediated gene delivery to the forefront of medical research (1). Viral vectors have the capacity for gene delivery into mammalian cells with high efficiency, but one of the crucial concerns in viral vectors is the immune response of the host. Another major concern is the ability of viruses to activate oncogenes and induce malignancies (2, 3). Hence, the development of efficient gene delivery systems with high safety is essential. Among the non-viral vectors in gene transfer, cationic polymers like PEI, Poly-L-Lysine (PLL), and dimethylaminoethyl methacrylate (PDMAEMA) have been extensively studied, due to the high efficiency of the gene delivery (4). Most of the cationic polymers used in gene delivery systems are non-biodegradable and so there is a risk of accumulation in the body, leading to complications (5, 6). Cationic polymers such as PEI have the ability to complex with RNA and DNA with high transfection efficiency (7). The electrostatic interaction between the positively charged amino groups on PEI and negatively charged phosphate groups on DNA causes the DNA to be neutralized and it be compacted (5). The disadvantages of using PEI polymer include high toxicity and low stability in gene delivery to the immune system (8, 9). Various methods were used to overcome these problems, including covalent binding of PEG to PEI, which increased the stability of nanoparticles in the blood circulation system. However, the presence of the PEG chain prevented suitable concentrations of DNA by creating steric hindrances for suitable interactions between PEI and DNA (10). The use of biodegradable polymers such as PLA has shown a great immense potential as scaffolds for tissue engineering and as a drug delivery carrier (11, 12). PLA is highly biocompatible, physically strong and have been extensively studied as delivery vehicle widely for proteins, drugs and other macromolecules such as RNA, peptides, and DNA (8, 13 and 14). PLA is one of the most popular biodegradable polymers due to its long clinical experience, unique degradation characteristics, and the ability to control the release of the drug. Many studies have shown that degradation of PLA could be employed for both clinical application and academic researchers (15). Moreover, it is possible to tune the rates of drug release from a few days to several years by controlling the molecular weight, by combination of PLA with other polymers and also by controlling ratio of each segment, the type of technique used to load the drug into nanoparticles, *etc.* (16-18). The large size of the particles obtained, the low efficiency of DNA encapsulation, and the inclination for hydrophobic interactions between plasma proteins and these polymers (which causes identification and elimination of the particles by the reticuloendothelial system) are among the obstacles preventing the use of the polymers in gene transfer systems 917, 19). The surface modification of hydrophobic polymers, such as PLA, by the hydrophilic polyethylene glycol, and the production of the amphiphilic polymer PLA-PEG polymer, can reduce the size of the resultant particles (because of the increased hydrophilicity) and also increase DNA encapsulation and duration of circulation in the blood compared with nanoparticles made of PLA alone (17, 20). The ability of PLA-PEG copolymer to mediate drug delivery into a wide range of eukaryote cell lines has been reported (21, 22). However, there are only a few studies on potential capability of PLA-PEG copolymer for gene delivery to eukaryote cells (19, 23). This was due to the electrostatic repulsion between the negatively charged phosphate groups of DNA and the carboxyl group of PLA as the resulted PLA-PEG could not neutralize the negative charges of DNA phosphate groups and reduced the gene delivery efficiency (24, 25). While the effects of simultaneous use of PLA-PEI-PLA with PLA-PEG-PLA on the DNA encapsulation, micelle stability, release kinetics and cell viability have been previously investigated, to our best knowledge no study has done to evaluate the effects of simultaneous use of PEI with PLA-PEG-PLA on the stability of the DNA in digestion buffer and gene delivery efficiency in serum-containing media (8, 24). Given the significant effect of PEI concentration on physicochemical properties and gene delivery efficiency of the proposed nanoparticles, the effect of the different mass ratio of PEI: (PLA-PEG-PLA) (w/w%) in PLA-PEG-PLA/PEI/DNA nanoparticles on physicochemical properties, DNA release rate, and gene delivery efficiency were first assessed. Given the high efficiency of the PEI polymer in gene delivery to mammalian cells, and also considering biocompatibility, biodegradability properties of PLA-PEG copolymers, in the present research work we propose to develop nano-carriers composed of PEI, PLA, and PEG polymers for efficient gene delivery into mammalian cells.

## Experimental

*Materials*

L-lactic acid, toluene, ethanol, ethyl acetate, antimony trioxide (Sb2O3), dichloromethane (DCM), polyethylene glycol (PEG 2000), Agarose gel, PVP, MgCL_2_, Tris-HCl, and EDTA were purchased from Merck (Germany) while stannous octoate, MTT, DMSO, RPMI 1640, and fetal bovine serum (FBS) were purchased from Sigma-Aldrich (USA). PEI was purchased from Polysciences (USA), DNase I from Polyscience and Thermo Fisher Scientific (USA), and MCF-7 breast cancer cell line and pEGFP-N1 were obtained from Pasteur Institute of Iran (PII).

*Synthesis of L-lactide*

L-lactide was synthesized from L-lactic acid, using antimony trioxide (Sb_2_O_3_) as a catalyst. The process comprised two steps, including oligomerization at 180–200 °C and then depolymerization and dimerization at 250 °C. In order to obtain more purity, the lactic acid was crystallized (three times) with ethyl acetate and dried in vacuum at 40 °C overnight before use.

*Synthesis of PLA–PEG–PLA copolymer*

**Figure 1:**
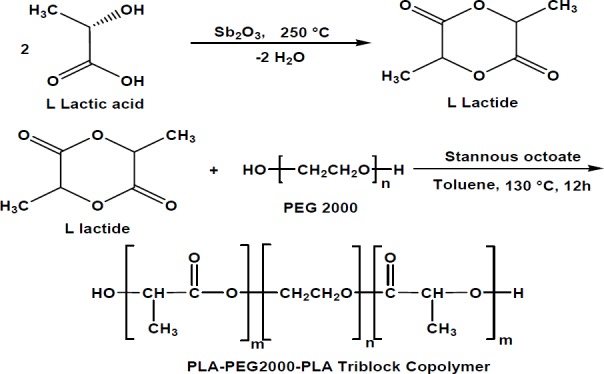
Schematic synthesis of L lactide and PLA-PEG-PLA triblock copolymers

The PLA–PEG–PLA copolymer was synthesized using ring-opening polymerization of D, L-lactide with PEG as an initial molecule and stannous octoate as a catalyst (Figure 1). Briefly, appropriate amounts of D, L-lactide, PEG, and Sn(Oct)_2_ were heated at 120 °C to start the polymerization. After 11 h, the polymer was cooled to room temperature, dissolved in chloroform, and precipitated in ethyl ether. The copolymer was dried under vacuum at room temperature for 24 h before use (26).

*Preparation and characterization of PLA-PEG-PLA/DNA and PLA-PEG-PLA/PEI/ DNA nanoparticles*

**Figure 2:**
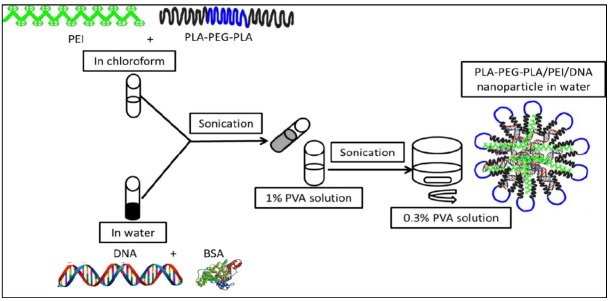
Encapsulation of plasmid DNA into PLA–PEG-PLA copolymer by double emulsion solvent evaporation technique

PLA-PEG-PLA/DNA was prepared using the double emulsion solvent evaporation technique (Figure 2) (17). An aqueous solution of BSA (2 mg) and 250 μg DNA (plasmid pEGFP-N1) were emulsified at 0 ºC for 30 sec (Sonicator® XL, Misonix, NY) containing 30 mg of PLA-PEG-PLA in 1 mL of chloroform. Subsequently, the mixture was emulsified in 1.5 mL of 1% w/v PVA solution at 0 ºC for two min. The emulsion was poured into 25 mL 0/3% PVA solution under moderate magnetic stirring. In order to remove chloroform, the emulsion was stirred at high speed for three hours at room temperature. Nanoparticles were collected by centrifugation at 16602×g for 2 h (Sigma 1-14K), following washing for three times with sterile distilled water to remove unentrapped PVA, PEI, DNA, and BSA, then the nanoparticles were lyophilized (Lifilizator Alpha model 1-2 LD plus). PLA-PEG-PLA/PEI/DNA nanoparticles were prepared as described above, except the PLA-PEG-PLA solution in chloroform, which also contained different mass ratios of PEI (w/w%) (1:300, 5:300, 10:300, and 15:300). The surface morphology of nanoparticles was examined by scanning electron microscope (LEO 1430, Zeiss, Oberkochen, Germany). The particle size and zeta potential were measured by dynamic light scattering (DLS) (Malvern Instruments, Westborough, MA, USA).

*Encapsulation efficiency*

The encapsulation efficiency of DNA in nanoparticles was determined by measuring the amount of DNA that was not encapsulated in nanoparticles. Therefore, the amount of DNA in the supernatant upon centrifugation of the nanoparticle suspension was measured by nanodrop spectrophotometers at 260 and 280 nm (Thermo Scientific 2000, USA) [DNA]f, then compared with the DNA used in the encapsulation process [DNA]t (28). The encapsulation efficiency was determined by the following equation: 

Encapsulation efficiency% = $\frac{\left[\mathrm{DNA}\right]t-\left[\mathrm{DNA}\right]f}{\left[\mathrm{DNA}\right]t}$ × 100

*DNase I and ultrasound protection assays*

The protective effects of PLA-PEG-PLA/PEI/DNA against DNase I was investigated using 0.8% agarose gel electrophoresis. The PLA-PEG-PLA/DNA and PLA-PEG-PLA/PEI/DNA nanoparticles at different mass ratios of PEI: (PLA-PEG-PLA) (w/w%) (1:300, 5:300, 10:300, and 15:300), were incubated with the naked DNA (the amount of DNA was 5 µg for each nanoparticle and naked DNA) in the presence of DNase I (1 U/μg of DNA) in a digestion buffer (Tris-HCL 50 mM and MgCl_2_ 10 mM) at 37 °C for 30 min. Then the DNase I was made inactive by adding 10 μL EDTA solution (0.25 M, pH 8.0). The nanoparticles were disassembled by adding heparin solution (1 w/v%) final concentration) and then incubated in a shaking incubator (120 rpm) for four hours at 42 °C (27). The samples with untreated DNA as a reference were analyzed by gel electrophoresis. Electrophoresis was performed on a 0.8% agarose gel at a constant voltage of 80 V for 2 h in the 1 × TAE buffer. To test whether nanoparticles can protect the loaded DNA from ultrasound waves, the nanoparticles contain 5 μg of DNA and naked DNA (5 µg) was treated with ultrasound (60 W for 30 min). The nanoparticles were disassembled and then analyzed by gel electrophoresis.

*DNA release assays*

One mg of PLA-PEG-PLA/DNA and PLA-PEG-PLA/PEI/DNA nanoparticles containing different mass ratios of PEI: (PLA-PEG-PLA) (w/w%) (1:300, 5:300, 10:300 and 15:300) was suspended in 1 mL of phosphate-buffered saline (PBS) at 37 °C with continuous shaking (100 rpm). The sample was centrifuged at 16,602 g for 1 h at regular intervals (1, 3, 7, 14, 21 and 28 days). The supernatant was then collected and subjected to DNA release analysis. The samples were resuspended in 1 mL of fresh PBS and incubated at 37 °C with continuous shaking (100 rpm). In order to assess the DNA release profile from nanoparticles, the amount of DNA in each sample was determined by NanoDrop spectrophotometers at 260 and 280 nm (28). The cumulative DNA release from the nanoparticles was calculated using the following equation: 

Cumulative release percentage% = $\frac{\mathrm{Total DNA content in supernatant of the PBS}}{\mathrm{The amount of DNA encapsulated into nanoparticles}}\times \mathrm{100}$

*Cytotoxicity studies*

The cytotoxicity of PEI, PLA-PEG-PLA copolymer, PLA-PEG-PLA/DNA, and PLA-PEG-PLA/PEI/DNA nanoparticles with the mass ratio of PEI: (PLA-PEG-PLA) (w/w%) of 15:300 was assessed by MTT assay in MCF-7 cells. MCF-7 cells were seeded in a 96-well plates at a cell density of 7,000 cells per well in 200 μL of complete media (RPMI 1640 medium supplemented with 10% FBS and antibiotics), and then incubated at 37 °C in 5% CO_2_. After 24 h, the cells were incubated with different concentration of PEI (5, 10, 20, 30 and 50 µg/mL), PLA-PEG-PLA copolymer, PLA-PEG-PLA/DNA and PLA-PEG-PLA/PEI/DNA nanoparticles containing PEI at concenterations of 100, 200, 400, 600, and 1000 µg/mL at 37 °C. Thereafter, MTT solution (20 µL, 5 μg/mL in culture medium) was added to each well and incubated for four hours at 37 °C. The supernatants were then removed and the formazan crystals were dissolved by DMSO (200 µL per each well). After incubating for 30 min at 37 °C, the optical density was measured at 570 nm by a microplate reader (BioTek Instruments; Winooski, VT, USA) (29). Finally, %viability was determined by the following equation:

viability% =$\frac{\left(\mathrm{absorbance of treated cells}\right)}{\left(\mathrm{absorbance of control cells}\right)}\times 100$

*In-vitro gene delivery*

MCF-7 cells (2 × 10^4^) were seeded into 24-well plates and cultured in 1 mL of complete medium at 37 ºC. After 24 h, the culture medium was replaced with 1 mL of complete medium containing 2 µg DNA in (PLA-PEG-PLA)-DNA and PLA-PEG-PLA/PEI/DNA nanoparticles at different mass ratios of PEI:(PLA-PEG-PLA) (w/w%) (1:300, 5:300, 10:300 and 15:300 of PEI into PLA-PEG-PLA). PEI-DNA complex was prepared by mixing plasmid DNA (pDNA) with PEI at N: P ratio of 5 (2 µg of DNA per well) and used as positive control. Moreover, 2 µg naked DNA was used as a negative control. After the 7 h incubation of the cells at 37 °C , the cell supernatant was replaced with 1 mL of fresh new complete medium and the cells were incubated for additional 48 h under the same conditions as described above (30). The expression of the green fluorescent protein (GFP) in cells was detected by the fluorescence inverted microscope (Nikon TE200). Transfection efficiency was also quantified by flow cytometry (CyFlow Space, Germany) and the data were analyzed with FloMax software.

*Statistical analysis*

At least three replications were used for all quantitative factors studied in the present research. Statistical analysis was performed using SPSS One-way ANOVA. The Kolmogorov-smirnoff test was employed to check the data set for normality, and Duncan’s multiple-range test was used at the 5% level to compare the means. The average of each treatment was then expressed together with the standard deviation (Mean ± SD). 

## Results and Discussion

*H-NMR results*

**Figure 3:**
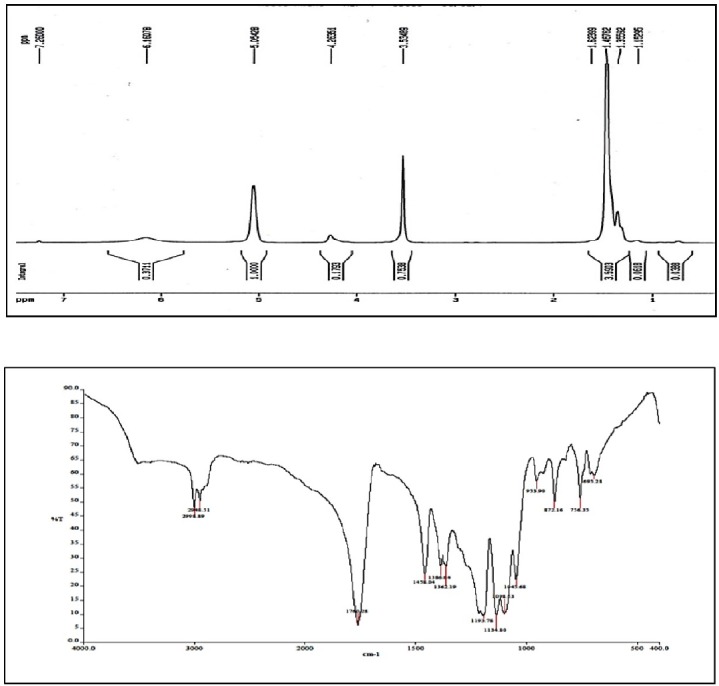
(A) HNMR and (B) FTIR spectrum of PLA-PEG-PLA copolymer

The peaks observed in 1.5, 5.2, and 3.6 ppm areas of H-NMR spectrum represent CH and CH_3_ groups in PLA tract and CH_2_ protons in PEG tract, respectively. Furthermore, the covalent bond between LA and PEG was confirmed by the present weak peak with several branches around the area of 4.3 ppm corresponding to the PEG-acylated protons of methylene units (Figure 3A).

*FTIR results*

The FT-IR spectrum of PLA-PEG-PLA copolymer is illustrated in Figure 3B. The absorption peak in 2900–3000 cm^-1^ area corresponds to C-H stretching of CH_3_ groups. Strong absorption peak in the region of 1760 cm^-1^ is related to the functional group of C=O. The peaks that appeared in the regions of 1190 cm^-1 ^and 1458 cm^-1^ are related to stretching C-O and the bending of -CH_2_- groups respectively. Also the peak in the 3500 cm^-1^ area is related to stretching of OH groups. Therefore, IR spectra of PLA-PEG-PLA copolymer confirms that the reaction between lactic and polyethylene glycol has been occurred (Figure 3B).

*Physicochemical Characterization of PLA-PEG-PLA/DNA and PLA-PEG-PLA/PEI/DNA nanoparticles*

**Figure 4:**
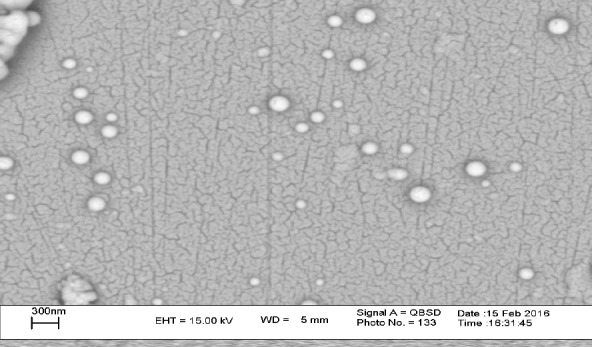
SEM image of PLA-PEG-PLA/PEI/DNA nanoparticles were prepared at PEI: (PLA-PEG-PLA) (w/w %) ratio of 15:300

A key factor in transfection efficiency and drug release kinetics is controlling the morphology of nanoparticles (18, 31). SEM images of PLA-PEG-PLA/PEI/DNA nanoparticles with PEI: (PLA-PEG-PLA) (w/w%) ratio of 15:300 were prepared. The SEM image showed that the PLA-PEG-PLA/PEI/DNA nanoparticles have a smooth surface and spherical shape (Figure 4). Next we measured the particle size and zeta potential of formulated nanoparticles by dynamic light scattering (DLS). The particle size and zeta potential are two important characteristics of nano-carriers, which play determinant roles in their biological half-life (32). Previous studies have shown that nanoparticles less than 0.5 µm can escape from recognition by the reticuloendothelial system (RES) which can lead to dramatic reduction in biological half-life of nanoparticles after intravenous administration (33, 34). The *in-vitro* investigations have shown that nanoparticles less than 1 µm have several times higher intracellular uptake as compared to larger microparticles (35). Figure 4 showed that the mean diameter and zeta potential of the samples varied, depending on the PEI concentration. Our results show that the mean particle size of nanoparticles increases by enhancing the PEI concentration in the formulations. The mean particle size of triplicates of PLA-PEG-PLA/DNA and PLA-PEG-PLA/PEI/DNA nanoparticles prepared at ratio of PEI: (PLA-PEG-PLA) (w/w%) (1:300, 5:300, 10:300 and 15:300), were 280 ± 19.76, 305.97 ± 10.74, 355.13 ± 14.96, 391 ± 14.34, 417.5 ± 5.21 nm respectively. Win *et al.* demonstrated that 100 nm nanoparticles had higher cellular uptake compared to smaller or larger nanoparticles (50, 500, and 1000 nm). They also found, although particles of 500 nm in size had less cellular uptake compared to the nanoparticles with 100 nm in size (1.3 fold), but these particles had higher cellular uptake compared to that 50 and 1000 nm particles (36). In some study, *in-vivo* biodistribution results of nanoparticles with different average particle sizes indicated that nanoparticles with an approximate size of 400 nm have a higher level of agglomeration in the lung, spleen, kidney, and liver (32). Therefore, regarding the particles size of PLA-PEG-PLA/PEI/DNA nanoparticles at the above mass ratio were 391 and 417 nm, respectively. It seems that these nanoparticles could be used for gene delivery to the lung, spleen, kidney, and liver.

**Figure 5:**
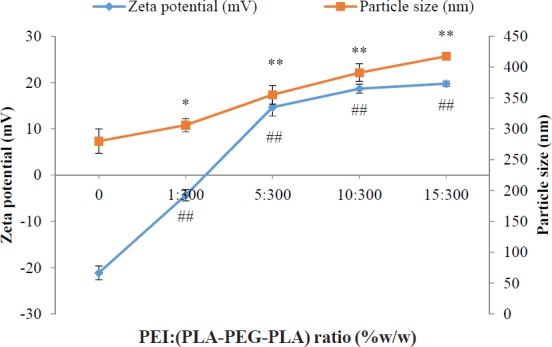
Effect of different mass ratios of PEI: (PLA-PEG-PLA) (w/w %) on particle size and zeta potential of PLA-PEG-PLA/PEI/DNA nanoparticles measured in pH 7.4. PEI: (PLA-PEG-PLA) (w/w %) ratios were from 0 PLA-PEG-PLA/DNA to 15:300 PEI: (PLA-PEG-PLA) (w/w %) in PLA-PEG-PLA/PEI/DNA nanoparticles (Error bars show ± standard deviation (SD), n = 3, ^*^ and ^#^*P* < 0.05, ^**^ and ^##^*P* < 0.01 compared with PLA-PEG-PLA/DNA nanoparticles)

**Figure 6:**
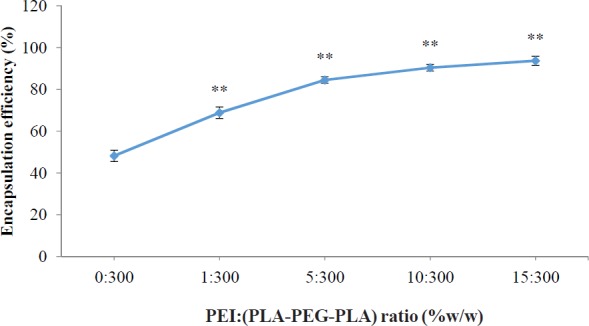
Effect of different mass ratios of PEI: (PLA-PEG-PLA) (w/w %) on DNA encapsulation in PLA-PEG-PLA/PEI/DNA nanoparticles. PEI: (PLA-PEG-PLA) (w/w %) ratios were from 0 (PLA-PEG-PLA)-DNA to 15:300 PEI: (PLA-PEG-PLA) (w/w %) in PLA-PEG-PLA/PEI/DNA nanoparticles (Error bars show ± standard deviation (SD), n = 3, ^**^P < 0.01 compared with PLA-(PLA-PEG-PLA)-DNA nanoparticles)

It has been reported that zeta potential is a very important key to determine the cellular uptake efficiency, and the *in-vivo *fate of nanoparticles (32). However, the optimum surface charges (*e.g.* negative, neutral or positive) and surface charge densities were reported differently for different types of drug delivery systems, in order to prolong plasma circulation time of nanoparticles, minimize the nonspecific binding of nanoparticles and prevent their loss to nontarget locations. For example, Yamamoto *et al.* (37) demonstrated that negatively charged PEG-PDLLA nanoparticles exhibited no significant difference in nanoparticles blood clearance kinetics; however, the negative surface charged of nanoparticles remarkably reduced the nonspecific uptake by spleen and liver, which was due to the electrostatic repulsion between negatively charged plasma membrane of the cells and nanoparticles. Conversely, Juliano *et al.* reported that the positively charged nanoparticles were cleared less rapidly compared to negatively charged ones, which was attributed to the tendency of negatively charged nanoparticles to coalesce in the presence of calcium ion and proteins in blood plasma (38). However, the cationic surface charge of the nanoparticles is a required factor for DNA condensation and cellular uptake (39). Electrostatic interactions between positively charged nanoparticles and negatively charged cell membranes cause cellular uptake of nanoparticles through the endocytosis mechanisms (40). The use of cationic polymers for plasmid transfection into mammalian cells has been well studied. Previous studies indicate that conjugated cationic polymers to biodegradable polymers create an electrostatically favorable interaction between the nanocarriers and the nucleic acid that is an essential parameter for successful transfection (41). The cationic PEI could increase the zeta potential of the PLA-PEG-PLA/PEI/DNA nanoparticles in comparison with the (PLA-PEG-PLA)-DNA nanoparticles possibly due to the cationic charge of PEI. The surface charge of nanoparticles of PLA-PEG-PLA/PEI/DNA nanoparticles were found to be dependent on the PEI concentration. Increases in the PEI concentration in the nanoparticles leads to an increased zeta potential of PLA-PEG-PLA/PEI/DNA nanoparticles. The zeta potential analysis of the surface charge of the nanoparticles showed that when the mass ratio of PEI: (PLA-PEG-PLA) (w/w%) was increased from 0:300 to 15:300, nanoparticles that were initially negatively charged at approximately -21.13 mV became positively charged at +19.73 mV (Figure 5). The effect of PEI concentration on the DNA encapsulation in nanoparticles is shown in Figure 6. The efficiency of DNA encapsulation was observed to be generally low for PLA-PEG-PLA, without the use of PEI (48.19%). Our research showed that the addition of the cationic PEI to the solution of PLA-PEG-PLA dramatically improved the encapsulation efficiency of plasmid DNA encapsulated in the PLA-PEG-PLA/PEI/DNA nanoparticles. The highest DNA encapsulation efficiency by the PLA-PEG-PLA in the presence of PEI increased by about two-fold relative to that without PEI (93.72%) (Figure 6). Moreover, such an improvement was correlative with the increase of PEI concentration in the formulation. As a typical example of increasing the mass ratio of PEI: (PLA-PEG-PLA) from 1:300 to 10:300 (w/w%), encapsulation efficiency increased from 68.83% to 90.39% (Figure 6). One limitation of hydrophobic biodegradable polymers like PLA in drug delivery systems is the poor encapsulation efficiency of hydrophilic macromolecules, such as DNA (19). Plasmid DNA is susceptible to damage by ultrasound and acidic environments (42). Moreover, the electrostatic repulsion between the negatively charged phosphate groups of DNA and the carboxyl group of PLA reduces the encapsulation efficiency (43). The cationic PEI neutralized the negatively charged DNA, giving the cationic complex a better chance to interact with negatively charged PLA.

**Figure 7:**
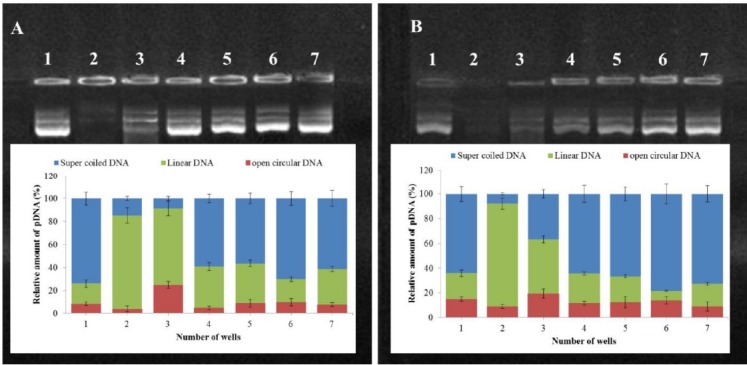
Stability of PLA-PEG-PLA/DNA and PLA-PEG-PLA/PEI/DNA nanoparticles containing different amounts of PEI against ultrasound waves (A) and DNase I (B); Lane 1: Naked plasmid DNA; Lane 2: Naked plasmid DNA after treatment in each lane description; Lane 3: (PLA-PEG-PLA)-DNA nanoparticles after treatment in each lane description; Lane 4-7: PLA-PEG-PLA/PEI/DNA nanoparticles containing different mass ratios of PEI: (PLA-PEG-PLA) (w/w %) (1:300, 5:300, 10:300 and 15:300) after treatment in each lane description. pDNA topology was quantified with ImageJ software )Error bars show ± standard deviation (SD), n = 3)

**Figure 8:**
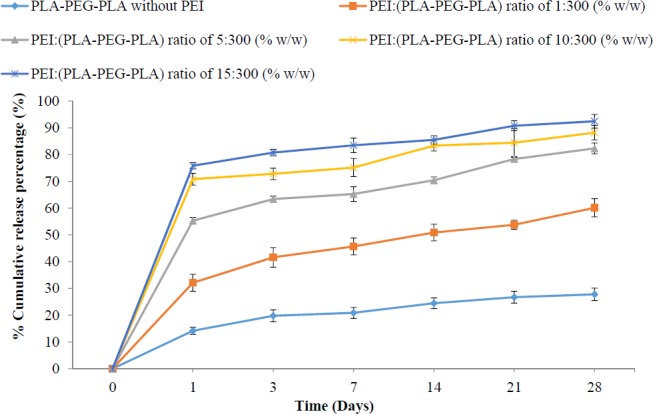
*In-vitro* release of plasmid DNA from PLA-PEG-PLA/PEI/DNA nanoparticles prepared with five different mass ratio of PEI: (PLA-PEG-PLA) (w/w %) at pH 7.4, (Error bars show ± SD, n = 3).

**Figure 9:**
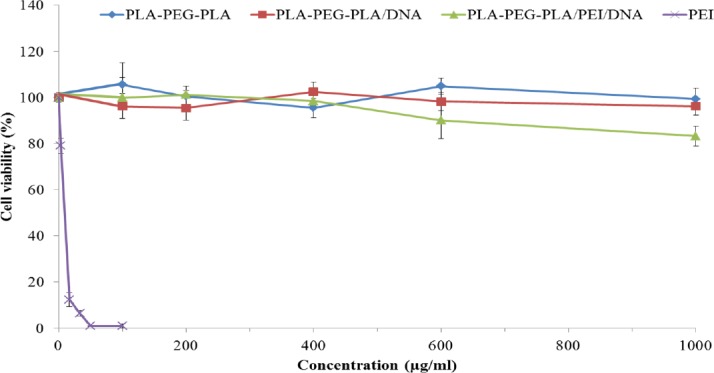
Toxicity of PEI, PLA-PEG-PLA copolymer, PLA-PEG-PLA/DNA and PLA-PEG-PLA/PEI/DNA nanoparticles at mass ratio of PEI: (PLA-PEG-PLA) (w/w %) of 15:300 on MCF-7 cells (Error bars show ± SD, n = 3)

**Figure 10:**
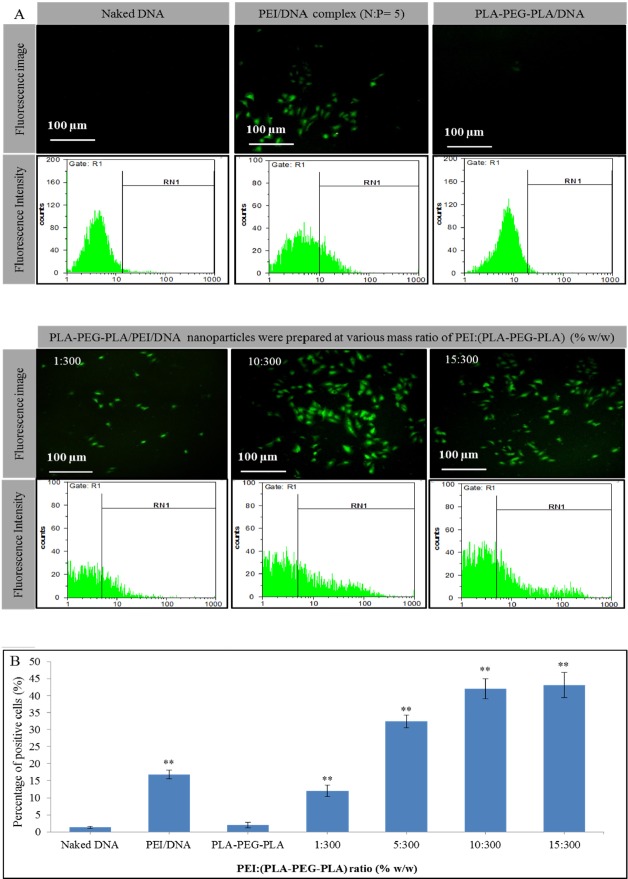
EGFP expression profile in MCF-7 cell line transfected by (A) naked DNA, PEI-DNA complex, PLA-PEG-PLA/DNA and PLA-PEG-PLA/PEI/DNA nanoparticles in the presence of 10% fetal bovine serum (DNA dose of 2 µg per well) (B) transfection efficiency in MCF-7 cells by PEI-DNA complex, PLA-PEG-PLA/DNA and PLA-PEG-PLA/PEI/DNA nanoparticles. (Error bars show ± SD, n = 3, ^**^P < 0.01 compared with naked DNA)

*DNase I and ultrasound protection assays*

A major barrier for gene delivery is the degradation of naked DNA by endonucleases, such as Deoxyribonuclease I (DNase I) or DNase I-like enzymes existing in the cellular cytoplasm and extracellular space (44-45). The effective condensation and covering of DNA by nanoparticles is an important factor for DNA stability against degradation by ultrasound damage and nucleases (46). To investigate protective ability of the (PLA-PEG-PLA)-DNA and PLA-PEG-PLA/PEI/DNA for DNA encapsulated within the nanoparticles against degradation by ultrasound damage and nucleases, the nanoparticles were exposed to DNase I and ultrasound waves. 

Agarose gel electrophoresis was performed to investigate whether the (PLA-PEG-PLA)-DNA and PLA-PEG-PLA/PEI/DNA could be stable in ultrasound damage and nuclease digestion. Figure 7 indicates that DNA recovered from the nanoparticles after incubation with DNase I and ultrasound, remained intact, while the naked DNA was completely digested after incubation with DNase I and ultrasound, as confirmed by the invisibility of the plasmid DNA bands in the agarose gel. This result demonstrates that PLA-PEG-PLA nanoparticles could protect the encapsulated DNA from ultrasound damage and nuclease digestion. Moreover, densitometric quantification of plasmid DNA bands showed that the DNA recovered from (PLA-PEG-PLA)-DNA nanoparticles after treatment with DNase I and ultrasound presented more of the open circular and linear forms, whereas the DNA recovered from PLA-PEG-PLA/PEI/DNA nanoparticles after treatment with DNase I and ultrasound were more supercoiled plasmid DNA (scDNA) forms regardless of the PEI concentration. Similar bands were observed for the control plasmid DNA demonstrating that coating DNA by PLA-PEG-PLA prevents the degradation of DNA by DNase I and ultrasound. Moreover, simultaneous encapsulated DNA and PEI into PLA-PEG-PLA copolymer prevent the conversion of the scDNA to open circular and linear forms.

Our study showed that DNA encapsulated in PLA-PEG-PLA protects DNA against digestion by ultrasound and endonuclease. Moreover, simultaneous encapsulation of DNA and PEI by PLA-PEG-PLA copolymers leads to improved protection of DNA. This is one of the important factors for efficient gene delivery by nanocarriers or ultrasound under *in-vitro* and *in-vivo* conditions.

*In-vitro release profile of DNA from (PLA-PEG-PLA)-DNA and PLA-PEG-PLA/PEI/DNA nanoparticles*

Some drugs have short plasma half-life therefore, several injections are required during the course of therapy which can have detrimental effects such as local tissue necrosis, pain, nerve damage, tenderness, and poor patient compliance (47). There are many alternative methods to reduce injection therapy, such as buccal (48, 49) oral (50-53), pulmonary (50, 54), and nasal 55-57) but these methods are not successful for clinical application of some drugs. To resolve these issues, scientists have focused specifically on the controlled release drug delivery systems based on biodegradable nanoparticles. The ability of copolymers containing PLA and PEG in controlling drug release has been demonstrated in several studies (8, 58).

The results showed that DNA release from (PLA-PEG-PLA)-DNA and PLA-PEG-PLA/PEI/DNA nanoparticles at first bursts, and then occurred slowly. Figure 8 shows, more than 50% of the released DNA over a period of 28 days occurred in the 24 h early. Similar studies in the past revealed that the reason for the burst of the release of DNA is the release of DNA in the surface of particles than encapsulated DNA in the core of particles. According to the comparison of averages DNA release from PLA-PEG-PLA/DNA and PLA-PEG-PLA/PEI/DNA nanoparticles, greater percentage of DNA encapsulated is released as the mass ratio of PEI in PLA-PEG-PLA/PEI/DNA nanoparticles increased. Hence, the lowest percentage release of encapsulated DNA in PLA-PEG-PLA/DNA nanoparticles was 27.78% with the mass ratio of PEI: (PLA-PEG-PLA) (w/w%) of 1:300 and the highest percentage of encapsulated DNA in PLA-PEG-PLAPEI/DNA nanoparticles with the mass ratio of PEI: (PLA-PEG-PLA) (w/w%) of 15:300 was about 92.45%. The results showed that the percentage of the burst release (first 24 h) increased and the percentage of slow release of DNA fell as the mass ratio of PEI in PLA-PEG-PLA/PEI/DNA nanoparticles increased. Several studies have indicated that the simultaneous use of a hydrophilic polymer with a hydrophobic polymer, leads to an increased release rate of the drug compared to hydrophobic polymer along (59). Formation of water channels within the matrix by hydrophilic polymers has been reported as one of the factors increasing the release rate of DNA (60). The *in-vitro* release profile of DNA from PLA-PEG-PLA/DNA and PLA-PEG-PLA/PEI/DNA nanoparticles indicated that there is a significant relationship between the mass ratio of PEI: (PLA-PEG-PLA) (w/w%) in PLA-PEG-PLA/PEI/DNA nanoparticles. Thus, this can lead to a change in the release profile of DNA from PLA-PEG-PLA, via a change in the mass ratio of PEI: (PLA-PEG-PLA) (w/w%) in PLA-PEG-PLA nanoparticles (Figure 8).

*PLA-PEG-PLA/PEI/DNA nanoparticles cytotoxicity*

The cytotoxicity of some nanocarriers is a major obstacle for gene delivery systems, therefore the evaluation of the cytotoxicity of the nanocarriers is important. As shown in Figure 9, PLA-PEG-PLA copolymer, (PLA-PEG-PLA)-DNA and PLA-PEG-PLA/PEI/DNA nanoparticles showed almost no cytoxicity (Cell viability was higher than 83%) in MCF-7 cells. However, free PEI was found to be highly toxic to the MCF-7 cells at concentrations higher than 5 µg/mL (Figure 9). Our study indicates that the PLA-PEG-PLA/PEI/DNA nanoparticles is a safe carrier, which can be due to the incorporation of PEI into the encapsulated PLA-PEG-PLAPEI/DNA nanoparticles. Some researchers have also reported similar results (60, 61). For example, Alshamsan *et al.* reported that the incorporation of PEI into PLGA nanoparticles, significantly improves the cytoxicity profile of PEI (60). The participation of PLA-PEG-PLA in preventing the surface exposure of the cationic charge of PEI, and thereby preventing the PEI-induced membrane disintegration, can be one of the reasons for the reduction in the cytoxicity of PLA-PEG-PLA /PEI-DNA nanoparticles compared with free PEI.

*In-vitro Transfection Studies*

Transfection of uncoated DNA into a cell is difficult due to some reasons, including large size, unsuitable surface potential, and early DNA digestion by the cell defense mechanism during intercellular and cell transfection (34, 62). In this study, transfection ability of nanoparticle was performed in the presence of RPMI 1640 medium supplemented with 10% FBS to verify whether the PEG, could increase gene delivery efficiency by preventing absorption of FBS proteins on the surface of the nanoparticles. The optimum time for protein expression depends on several factors such as gene transfer method, scale and type of cells, vector used for expression, protein half-life, and *etc*. (63-65). A similar study found that the highest transfection efficiency of GFP into MCF-7 cells was observed at 24-48 h post-transfection (65) since the plasmid DNA gets lost after a few cell cycles, increasing post-transfection time, leading to the over proliferation of cells (without plasmid) and hence resulting in lower gene transfer efficiency (66). Moreover, Zhang *et al.* reported that there were no significance difference between 24 h and 48 h post transfection (65). The DNA release profile results indicated that the highest DNA release ratio of nanoparticles, wasobserved after 24 h incubation in PBS. Therefore with regard to the previous studies and DNA release profile from PLA-PEG-PLA/PEI/DNA nanoparticles, the appropriate time period for post transfection is selected 48 h.

Transfection efficiency of PLA-PEG-PLA/PEI/DNA nanoparticle were investigated in comparison with PEI-DNA complexes at N/P ratio of 5. The ability of nanoparticles in the transfection of DNA into MCF-7 cells has been proven by fluorescence microscopy and flow cytometry (Figure 10). A fluorescence microscopy image showed a green emission in some MCF-7 cells treated with PLA-PEG-PLA/PEI/DNA nanoparticles. These results demonstrate the ability of the nanoparticles to transfer and intracellular release of DNA into the MCF-7 cells. However, the naked DNA in a serum-containing medium was unable to transfect MCF-7 cells. In the case of the PLA-PEG-PLA/DNA nanoparticles, very low expression of GFP was observed and the expression was similar to the negative control group (naked pDNA). As mentioned above, the positive surface charge of nanoparticles is essential for gene delivery into cells (39). It seems that this low expression of GFP in PLA-PEG-PLA/DNA nanoparticles is due to the inability of PLA-PEG-PLA copolymer to neutralization negative charges of DNA phosphate groups (25). The transfection efficiency of PLA-PEG-PLA/PEI/DNA nanoparticles were prepared with different mass ratio of PEI: (PLA-PEG-PLA) (w/w%) (1:300, 5:300, 10:300, and 15:300 (, was 12.03, 32.29, 42.01, and 43.08 percent respectively (Figure 9). In this study, PEI-DNA complex was used as positive control. PEI is one of the most potent transfection reagents that use as non-viral gene delivery systems, but the toxicity and non-biodegradability of PEI have restricted its use in clinical applications. The efficiency of gene expression in MCF-7 cells that was treated with PEI-DNA complex, was 16.76 percent. Although PLA-PEG-PLA/PEI/DNA nanoparticle was prepared at 1:300 ratio of PEI: (PLA-PEG-PLA) (w/w%), shows lower transfection efficiency compared to PEI-DNA complex, increasing the ratio concentration of PEI in PLA-PEG-PLA/PEI/DNA nanoparticles significantly improved the GFP expression (Figure 10).

Although nanoparticle surface charge is another important factor in gene delivery efficiency and DNA condensation, but higher cytotoxicity of cationic nanocarriers have been reported (67, 68). Moreover, cationic nanovectors such as PEI and cationic liposomes tend to absorb proteins and polyanion in the plasma. Interactions between nanoparticles and serum compounds leading to low stability of polycation-DNA and early release of DNA in these conditions (8). Moreover, these phenomena increases the particle size of polycation-DNA, and as a result it will be difficult for the complex to pass across the cell membrane. The interaction of the polycation-DNA with plasma proteins allows the immune system to identify and eliminate polycation-DNA quickly (8, 19). That’s why in most studies, serum-free medium (medium without FBS) has been used to investigate gene transfer efficiency (69, 70). To reduce protein binding to the cationic surface of nanoparticles, hydrophilic polymers such as poly (ethylene glycol) (PEG) have been coupled to cationic nanoparticles (71). PEG as an exterior shell in the nanoparticles prevents non-specific interactions with serum and minimizes accumulation of particles (72). Our results indicated that PEI-DNA coating by PLA-PEG-PLA copolymer leads to the decrease of PEI-DNA cytotoxicity on MCF-7 cells. Moreover, the present study indicated that PLA-PEG-PLA/PEI/DNA nanoparticles in a serum-containing medium have a great ability to transfer the gene to MCF-7 cells in comparison with PEI-DNA complexes. Similar results have also been reported by Chan *et al*. and Fu *et al.* (23, 73). Furthermore, another advantage of using copolymers containing hydrophobic and hydrophilic segments, is the ability of these copolymers to adjust drug release profiles by changing the molecular weight ratio of hydrophobic to hydrophilic segments in copolymer and, the method of preparation of nanoparticles, *etc.* (16, 17, 59 and 74). Hence, it seems that the use of PLA-PEG copolymers is desirable in the gene therapy of some diseases that require the sustained-release form.

## Conclusion

This research aimed to propose a multi-functional micellar system for gene transfer with the advantages of cationic, biodegradable, and amphiphilic polymers at the same time. The research results revealed that the simultaneous use of PEI and PLA-PEG-PLA polymers reduces PEI toxicity, improves DNA release from PLA-PEG-PLA copolymer, and protects DNA from the damage caused by ultrasound and enzymatic digestion. They also showed that PLA-PEG-PLA/PEI-DNA nanoparticles have a great ability to transfer the gene into MCF-7 cells, as compared with the PEI/DNA complex in serum-containing media.
